# The role of psychological stress in the pathogenesis of psoriasis

**DOI:** 10.3389/fmed.2025.1614863

**Published:** 2025-08-11

**Authors:** Dongyun Lei, Canyi Gong, Bin Wang, Litao Zhang, Guoqiang Zhang, Mao-Qiang Man

**Affiliations:** ^1^Department of Dermatology, Tianjin Academy of Traditional Chinese Medicine Affiliated Hospital, Tianjin, China; ^2^Dermatology Hospital of Southern Medical University, Guangzhou, China; ^3^Department of Dermatology, The First Hospital of Hebei Medical University, Shijiazhuang, China; ^4^Hebei Technical Innovation Center for Dermatology and Medical Cosmetology Technology, Shijiazhuang, China

**Keywords:** psoriasis, psychological stress, psoriasis area and severity index, intervention, PSAI

## Abstract

Psoriasis is an immune-mediated dermatosis characterized by systemic inflammation and multifactorial pathogenesis. Among its many triggers, psychological stress has emerged as a pivotal yet underappreciated contributor to disease onset and exacerbation. Although the pathomechanisms by which psychological stress is involved in the pathogenesis of psoriasis are not clear, evidence suggests a regulatory role of psychologic stress in immune functions, including increasing expression levels of proinflammatory cytokines and intracellular adhesion molecule-1 (ICAM-1), and decreasing anti-inflammatory cytokines and the function of glucocorticoid receptors, possibly in part via activation of corticotropin-releasing hormone (CRH)-proopiomelanocortin (POMC)-adrenocorticotropic hormone (ACTH)-corticosteroids axis. In addition, the onset and/or worsening of psoriasis can also be attributed to psychological stress-induced defective epidermal permeability barrier function. Moreover, the bidirectional nature of this relationship often leads to a vicious cycle of flare-ups and psychological distress, further complicating patient management and quality of life. This review aims to synthesize current evidence on the relationship between stress and psoriasis, examining mechanistic pathways through which psychosocial stress contributes to immune dysregulation in psoriatic pathology. It also underscores the significance of psychological interventions in the management of psoriasis.

## 1 Introduction

Psoriasis is often comorbid with various health conditions. In addition to obesity, type 2 diabetes and cardiovascular disorder (1–3), psoriasis is also associated with psychological disorders. In murine, depression-like behavior is observed following induction of psoriasis-like dermatitis by imiquimod (4). In humans, the prevalence of mental disorders can be as high as 90% in psoriatic patients (5). Prevalence of psychological stress is higher in psoriatic patients than in the controls (6). The prevalence of depression can be as high as 74.6% in individuals with psoriasis depending on the geographic region (7). A meta-analysis showed that psoriasis increases the risk of depression with odds ratio (OR) of 1.57 and the pooled prevalence of depressive symptoms is 28% in individuals with psoriasis (8). The risk of developing depression is higher in patients with moderate-to-severe psoriasis than those with mild disease (9). Likewise, the severity of depression is higher in individuals with moderate–severe psoriasis than those with mild psoriasis (9). Although severe psoriasis (PASI ≥10) does not significantly increase the risk of depression measured by Hospital Anxiety and Depression Scale – Depression (HADS-D)(OR = 1.52, 95% CI: 0.89–2.61) (10), PASI is positively associated with Patient Health Questionnaire-9 (PHQ-9) (Standardized Beta Coefficient = 0.465, *p* < 0.05) (11). Madrid Álvarez et al. reported that both HADs-D and HADS-S were significantly higher in psoriatic patients than in non-psoriatic controls (12). Moreover, the prevalence of anxiety is also higher in individuals with psoriasis than those without psoriasis (odds ratio = 2.91, 95% CI: 2.01–4.21, *p* < 0.001) (13). The severity of anxiety (General Anxiety Disorder-7, GAD-7) is correlated positively with PASI (Standardized Beta Coefficient = 0.515, *p* < 0.05) (9). But HADS – Anxiety (HADS-A) does not differ significantly between psoriatic patients and normal controls (14), suggesting that HADS may not be as sensitive as GAD-7 and PHQ-9 in the assessment of some psychological conditions at least in psoriatic patients. Regarding the association of psoriasis and suicidality, the results are inconclusive. A population-based study in 149,998 psoriatic patients and 766,950 controls revealed that psoriasis increases the risk of suicidality with hazard ratio of 1.44 in USA (15). Similarly, a study in multiple European countries showed the adjusted OR of suicidality was 1.94 (95% CI, 1.33–2.82) in psoriatic patients (16). However, the results of a meta-analysis did not find an increased risk of suicidality in individuals with psoriasis (risk ratio = 1.26; 95% CI: 0.97–1.64) (17). Similar results were obtained in a study in 169,909 Taiwanese (adjusted hazard ratio = 1.17, 95% CI: 0.88–1.55) (18). Another lager population-based study, including 363,210 psoriatic patients and 1,801,875 controls, showed that only mild psoriasis increases the risk of severe mental illness, while moderate-to-severe psoriasis does not increase the risk of severe mental illness (19). Thus, further studies are needed to determine whether psoriasis increases the risk of suicidality, and the other conditions, such as smoking, body mass index and alcohol consumption, influence the development of psychological disorders in individuals with psoriasis.

In this review, we summarize the evidence of the link between psoriasis and psychological stress, and discuss the underlying mechanisms by which psychological stress negatively impacts psoriasis, based on the published literature searched via PubMed and Google Scholar from inception through January 2025.

## 2 The link between psychological stress and psoriasis

A bulk of evidence indicates a mutual influence of psychological stress and psoriasis. Psoriasis can increase the risk of psychological stress and vice versa. The risk of psoriasis correlates positively with the severity of psychological stress (20). On the other hand, the greater the Psoriasis Life Stress Inventory score (a measurement of psoriasis-related stress), the severer the psoriasis (21), suggesting proper management of one condition can benefit the other one.

### 2.1 Psychological stress triggers/exacerbates psoriasis

The etiology of psoriasis is obscure although several hypotheses have been proposed. However, a number of studies have demonstrated a pathogenic role of psychological stress in psoriasis. A questionnaire survey showed that 61% of psoriatic patients strongly believe the contribution of stress/psychological factors to the development of psoriasis (22). In agreement with this finding, a 9-year-old girl without family history of psoriasis developed psoriasis 3 weeks after being frightened by firecrackers (23). Similarly, a 54-year-old male suffered from psoriasis 6 months after engaging in homeland war (24). In comparison to normal controls, psoriatic patients more often have negative personal experiences of traumatic events such as emotional abuse from early childhood to adulthood (*p* < 0.01) (25) or higher Perceived Stress Scale, HADS-A and HADS-S prior to the onset of psoriasis (26). Adverse childhood experience scores are higher in psoriatic patients than in controls (*p* < 0.0001). Patients with early onset psoriasis experience more traumatic events than the controls (*p* < 0.01) (27). Over 54% of psoriatic patients have at least one potential stressful event whereas only 19.5% of the controls have stressful event (OR = 4.92). The rates of stressful events in new onset and recurrence patients are 47.36 and 63.51%, respectively (28). In a 3-year follow-up of psoriatic patients whose lesions were cleared, 39% of the relapsed cases experienced stress event one month prior to the relapse. Relapse of psoriasis can occur as soon as 2 days after being exposed to stress (29). Importantly, the levels of psychological stress are positively associated with psoriasis severity with pearson r = 0.28 (*p* < 0.05) (30). The role of psychological stress in the pathogenesis of psoriasis is also demonstrated in individuals with various resilience, an ability to cope with and recover from setbacks. In males, individuals with low stress resilience (9-point Standard Nine scales 1–3) have a higher risk of psoriasis with adjusted hazard ratio of 1.31 (95% CI, 1.26–1.36), compared with individuals with high stress resilience (9-point Standard Nine scales 7–9) (31). Likewise, the risk of psoriasis in higher in individuals with higher Holmes and Rahe Scale (>100) than in those with lower Holmes and Rahe Scale (32). Moreover, psychological stress negatively influences the therapeutical efficacy. High-level worry is a risk factor for delaying the clearance of psoriatic lesions in psoralen-UVA photochemotherapy, with a relative risk (ExpB) of 1.81 (33). Furthermore, evidence suggests a gender difference in response to psychological stress. For instance, females with psoriasis vulgaris and males with guttate psoriasis are more sensitive to stressful events (28). Female psoriatic patients have more adverse childhood experience than males (27). Additionally, gender-based differences in psychological stress responses are, in part, attributable to the modulatory effects of estrogens on hypothalamic–pituitary–adrenal (HPA) axis function. Previous studies demonstrated that subcutaneous injection of estradiol augments stress-induced increased in plasma levels of both cortisone and adrenocorticotropic hormone (ACTH), while inhibiting glucocorticoid-mediated negative feedback on stress-induced activation of the HPA axis (34, 35). Glucocorticoids can increase the production of chemokine ligand 20 (CCL20), consisting with the elevated CCL20 levels in patients with psychological disorders (36). The latter recruits Th17 cells to the skin, participating the development to psoriasis. These results probably explain, in part, a slightly higher prevalence of psoriasis in females than in males, particularly in individuals under 60 years old in some regions although other studies did not demonstrate gender-related differences in the prevalence of psoriasis (37–39). Taken together, a growing bulk of evidence indicates a pathogenic role of psychological stress in psoriasis.

However, scattered evidence suggests no relationship between psychological stress and psoriasis. A study in 16 patients with recent psoriasis worsening and 16 controls showed no differences in the number of life events between these two groups over the past 12 months (40). Other studies suggest that antidepressants may account for the increased prevalence of psoriasis in individuals with psychological disorders because the proportion of individuals with a history of taking antidepressants prior to onset of psoriasis is higher than those without a prior history of taking antidepressants (OR = 2.78, 95% CI: 0.964–8.105) (41). Consistent with this finding, antidepressant-induced psoriasis has been reported in several publications (42–46). Thus, further studies are needed to assess the link between psychological disorders and psoriasis.

### 2.2 Mitigation of psychological stress ameliorates psoriasis

Because of the potential pathogenic role of psychological stress in psoriasis, mitigation of psychological stress can be a valuable approach in the management of psoriasis. Indeed, several studies have demonstrated the benefit of psychological intervention for psoriasis although not significant improvement was observed in some studies (Table 1) (47–58). The lack of significant improvement may be attributable to the inclusion of patients with mild to moderate psoriasis, as greater clinical response was observed in those with a PASI score greater than 6 (47). Another study demonstrated that psychological intervention significantly improved anxiety compared to controls, while both groups showed comparable improvement in PASI scores. The authors attributed the PASI improvement in both groups to seasonal weather changes during the study period (49). Therefore, disease severity and seasonal weather conditions may influence the effectiveness of psychological interventions in patients with psoriasis.

**Table 1:** Influences of psychological intervention on psoriasis.

Type of psoriasis	Protocol	Outcome	References
No Effect on PASI			
Mild–moderate plaque psoriasis	The study included 33 controls and 26 cases in intervention group. Both groups received conventional therapy for 6 weeks. Patients in the intervention group were also given psychological intervention.	No significant differences in reductions in PASI (0.40 ± 1.06 vs. 0.56 ± 1.42, *p* = 0.619)	(47)
Moderate-to-severe plaque psoriasis	Among 42 patients who received conventional therapy for 6 months, 20 patients also received psychological therapy.	Changes in PASI did not differ significantly between the two groups (8.0 ± 6.4 vs. 7.1 ± 7.5, *p* = 0.63)	(48)
Mild–moderate plaque psoriasis	33 psoriatic patients were given a web-based psoriasis-specific CBT and 45 cases were without CBT	Changes in PASI did not differ significantly between the two groups (0.7 vs. 1.0, *p* = 0.67)	(49)
Plaque psoriasis	Psoriatic patients wrote emotional disclosure of either King type (*n* = 12) or Pennebaker type (*n* = 15) prior to each UVB therapy 3 times weekly for maximum of 8 weeks. 13 patients without writing emotional disclosure served as controls.	No significance in SAPASI among the groups at the end of the study	(50)
Psoriasis	39 psoriatic patients received usual treatment. But 18 patients in the mindfulness-based intervention (MBI) groups also received 8 sessions of MBI in 2 weeks. Both groups were followed up for 3 months.	Significant reductions in SAPASI were observed in control group (*p* = 0.035), but not in the MBI group (*p* = 0.06)	(51)
Improve PASI			
Moderate-to-severe plaque psoriasis	29 patients received conventional therapy, while 42 patients received combination of conventional therapy and psychological intervention. Both groups were followed 6 months after the end of therapy	Significant reductions in PASI were observed in combination therapy group (4.76 ± 6.46 vs. 1.62 ± 4.62, *p* < 0.05)	(52)
Psoriasis	Among 22 patients who received usual treatment, 9 patients were given 2 educational lessons per week for 12 weeks.	At 3 months, PASI was decreased from baseline 8.4 (95% CI 6.0–10.8) to 6.8 (95% CI 4.3–9.3), *p* < 0.05, in the intervention patients, while in the controls, PASI was decreased from 8.1(95% CI 5.8–10.4) to 7.1 (95% CI 4.8–9.4).	(53)
Psoriasis	In addition to usual medical treatment, 6 patients received mindfulness intervention for 8 weeks and 13 controls	Patient SAPASI was reduced from baseline 5.94 ± 3.94–3.65 ± 1.37 (*p* = 0.05) in the intervention group. But in the controls, patient-self assessment PASI remained no significant changes (7.65 ± 5.68 vs. 7.02 ± 5.53.)	(54)
Chronic plaque psoriasis	30 patients received medical treatment and 28 patients received both medical treatment and PSMP once weekly for 6 weeks and followed up for 6 months.	In comparison to medical treatment alone, combination of usual medical treatment and PSMP significantly lowered PASI at both 6 weeks (*p* = 0.03) and 6 months (*p* = 0.04).	(55)
Moderate to severe psoriasis	Among 21 patients receiving UVB therapy, 10 patients were given intervention with mindfulness-based stress reduction.	In comparison to UVB alone, addition of mindfulness-based stress reduction shortened the time of 50% lesion clearance (75 percentile 51 vs. 100 days, *p* = 0.002).	(56)
Psoriasis	83 patients received usual care and 79 patients received usual care and one motivational intervention, followed by 6 phone-based intervention in the next for 12 weeks	After 3 months, self-administered PASI was lower in motivational intervention than in the controls (−2.47, 95% CI – 3.94 to −1.00, *p* = 0.001).	(57)
Psoriasis vulgaris	23 patients received psychotherapy once weekly for 4 weeks, followed by once every 2 weeks for 3 times. 21 patients without psychotherapy served as the controls. All patients received no other treatment.	By the end of the study, 74% of patients in the intervention group and 43% in the controls showed reductions in PASI. 91% of patients in the intervention group showed improvement in total psoriasis sign score, while only 29% in the controls.	(58)

CTQ, childhood trauma questionnaire; LES, life events scale; NS, not specified; OR, odds ratio; HRS, Holmes and Rahe Scale; CBT, Cognitive behavioral therapy; PSMP, Psoriasis Symptom Management Programme; SAPASI, self-administered psoriasis area and severity index.

In addition, Schmid-Ott reported that a 48-year-old woman who had been suffering from psoriasis since childhood was successfully treated by depth psychology-founded psychotherapy. Later her psoriasis relapsed because of the death of her near relative. Again, her psoriasis was remarkably improved following psychotherapy (59). Another study in a small group of subjects (*n* = 18) with over 1-year history of psoriasis on the scalp showed that psoriasis severity was decreased by 27.5% (ranging from 0 to 48%) following 12-week meditation with or without imagery, whereas no significant changes were observed in the controls (60). Psoriasis Symptom Management Program (PSMP) is an approach to manage psychological conditions by providing patients with information about the medical and biological basis of psoriasis, and stress reduction techniques once weekly for 6 weeks (61). In comparison to regular medical treatment alone, combination of regular medical treatment with PSMP exhibits superior efficacy in the improvements in multiple aspects of psoriasis, including PASI (*p* = 0.03), HADS-A (*p* = 0.007), HADS-D (*p* < 0.001) and psoriasis life stress inventory scores (*p* < 0.001). The clearance of skin lesions >75% was observed in 64% of the patients in the combination group and 24% in the regular treatment group at 6-month follow-up (55). Correspondingly, Bostoen et al. reported that appropriate education program benefits psoriasis. In addition to medical treatment, psoriatic patients were also educated on the patient’s skin disease, a healthy lifestyle, application of stress-reducing techniques and feedback twice weekly for 12 weeks. Patients receiving medical treatment alone served as controls. Following 12-week intervention, more significant reductions in PASI, dermatology life quality index and psoriasis disability index were observed in the intervention group (*p* < 0.05 vs. controls for all). These benefits (except dermatology life quality index) sustained for 3 months after the end of intervention (53). Combination of conventional anti-psoriasis therapy with either Mebicar (antianxiety) or Mianserin (antidepressant) also significantly shortens the time to achieve PASI50 and PASI75 in comparison to conventional anti-psoriasis therapy alone (62). Similarly, a study in 10 patients showed that combination of antidepressant (fluoxetine) and PUVA therapy (psoralen + UVA) synergically improved psoriasis (63). A 5-year study demonstrated that antidepressants can lower the risk of psoriasis with adjusted hazard ratio of 0.69 (64). In addition, using relaxation/visualization techniques in conjunction with phototherapy or photochemotherapy treatments for an average of 18.9 treatment sessions can achieve 95% clearance of skin lesion, whereas 40 treatment sessions are required to achieve 95% clearance in psoriatic patients treated with phototherapy or photochemotherapy alone (65). Interestingly, psoriasis can be improved by active suggestions of patients that their psoriasis would be improved (66). Psychological intervention does not only improve psoriasis, but also prevents the recurrence. Seville reported that patients whose psoriasis was precipitated by stress were given psychological intervention by explanation of the negative influence of stress on psoriasis after complete clearance of their skin lesions, and followed up for 3 years. At the end of 3-year follow up, 74% of patients who understood and accepted the explanation remained skin lesion free. But only 20% of patients who did not understand and accept the explanation were skin lesion free (*p* < 0.0001) (67). Collectively, this bulk of evidence indicates the benefit of psychological intervention in the management of psoriasis.

## 3 Underlying mechanisms by which psychological stress negatively influences psoriasis

Psoriasis is an inflammatory dermatosis. Though inflammation increases risk of depression (68, 69), immobilization stress significantly increases the serum levels of proinflammatory cytokines IL-1β, IL-6, IFNγ, and monocyte chemoattractant protein-1 (MCP-1), while decreasing anti-inflammatory cytokines such as IL-10 (70). Psychological stress is positively associated with circulating levels of proinflammatory cytokines such as IL-1 and IL-6 in humans (71, 72). Evidence also suggests a pathogenetic role of psychological stress in inflammatory dermatoses (73, 74), including psoriasis, via divergent mechanisms.

### 3.1 Central and peripheral hypothalamic–pituitary–adrenal axis

Although ample evidence indicates psychological stress negatively impacts psoriasis, the underlying mechanisms are still obscure. But the involvement of “the Brain-the Skin Axis” has been widely speculated (75–77). This speculation is supported by a number of studies in humans and animals. Acute psychological stress stimulates the production and secretion of corticotropin-releasing hormone (CRH) in the pituitary portal circulation. The latter reaches the adenohypophysis, stimulating the release of ACTH into the circulation. At the cortical area of adrenal gland, ACTH stimulates secretion of adrenal glucocorticoids (78). Glucocorticoids exhibit ani-inflammatory property, ameliorating inflammation (79). However, psychological stress-induced increase in glucocorticoid can decrease the sensitivity of glucocorticoid receptor, compromising its anti-inflammatory effects, leading to the development of inflammation (80–82). On the other hand, peripheral HPA axis also exists in the skin (83) because both the skin and the brain are developed from the ectodermal germ layer during the embryo development (84). The keratinocytes can also secrete ACTH, *α*-MSH and *β*-endorphin, neuropeptides (85). Moreover, keratinocytes and fibroblasts express the receptors of corticotropin releasing factor (CRF), ACTH, proopiomelanocortin (POMC), CRH, and β-endorphin (86–89). The expression levels of mRNA for POMC, CRH receptor type 1, melanin-concentrating hormone receptor (MCHR1) and melanocortin receptors 2–4 are markedly elevated in both psoriatic lesional and non-lesional skin compared with non-psoriasis healthy controls (90), indicating the present of altered peripheral HPA in psoriatic individuals. In the skin, CRH activates CRH receptor 1, resulting stimulation of dermal fibroblast proliferation, while increasing expression levels of the interferon *γ*-stimulated hCAM and ICAM-1 adhesion molecules in keratinocyte cultures (91, 92). In addition, CRH stimulates production of corticosteroids in both melanocytes and fibroblasts (93–95). Excessive corticosteroids reduces feedback sensitivity of HPA axis (96), leading to a decrease in peripheral corticosteroids levels. Stress scores negatively correlate with cortisol levels (97). CRH induces degranulation of mast cells along with increases in vascular permeability and expression vascular endothelial growth factor (98, 99), the features of psoriasis. Further, salivary cortisol levels were lower in psoriatic patients who believed to highly respond to stress (*p* < 0.01 vs. patients whose psoriasis was unrelated to stress) (100). Additionally, stress induces a more dramatic increases in the number of monocyte and CD4(+) cells in comparison to the non-stressed controls (101), in addition to increases in cytotoxic CD8 + T lymphocytes and CLA + CD3 + lymphocytes (102). Coupling with reduced sensitivity to corticosteroids in chronic stress (103), stress can provoke and exacerbate inflammation.

As aforementioned, stress increases ACTH. The latter stimulates the release of epinephrine from adrenal gland. The binding of epinephrine to adrenergic-β2 receptors activates NFκB and ERK signaling pathways, increasing NF*κ*B transcriptional activity and/or release danger-associated molecular patterns, such as heat-shock proteins and adenosine triphosphate, resulting in an increase in circulating levels of proinflammatory cytokines, including IL-6 and IL-1β (104). Collectively, both brain-skin and peripheral HPA axis can contribute to stress-induced increases in proinflammatory cytokines, leading to the development and exacerbation of psoriasis (77, 105).

### 3.2 5-hydroxytryptamine

5-hydroxytryptamine (5-HT) is a neurotransmitter in both central and peripheral nervous systems, which is linked to several psychological conditions such as depression and psychosis (106). Both serotonergic neurons in the central nervous system and peripheral cells (gastrointestinal mucosa, adipocytes, T cells and mast cells) can synthesize 5-HT (107). Because 5-HT cannot pass through blood–brain barrier, 5-HT in extra central neural tissues is mainly from peripheral sources (108). About 95% of 5-HT in the body is from the gastrointestinal tract, with 90% from enterochromaffin cells (109). Although decreased 5-HT is proposed to be attribute to depression (110), some types of stress, such as immobilization and heat, increase 5-HT in the rat brain (111–113) and plasma (114). In addition, repeated forced swimming increases extracellular levels of 5-HT in the striatum of rat brain (115), and maternal separation stress in early life increases 5-HT in intestinal tissue and serum of mice (116). Some immune cells such as mast cells, T cells, neutrophils and monocytes express both 5-HT and its receptors (109). The binding of 5-HT to its receptor can attract inflammatory cells and increase the production of proinflammatory cytokines such as IL-1 and IL-6 via activation of NF*κ*B signaling pathway (109). Correspondingly, psychological stress increases expression levels of IL-1β, IL-6, and TNF-*α* in the intestine (111). 5-HT also stimulates the production of interferon *γ* by NK cells (107). Conversely, either reduction in 5-HT production or blockade of 5-HT action downregulates IL-1, IL-2, IL-6 and TNF-a, and inhibits T-cell activation (109). A clinical study showed that serum 5-HT levels are significantly higher in psoriatic patients than in non-psoriasis controls and positively correlated with PASI in patients with symptoms of either depression or anxiety (117–119). Similarly, psoriatic patients with anxiety exhibit higher serum levels of 5-HT (117). The expression levels of 5-HT are also higher in psoriasis-involved skin than in either the uninvolved skin or normal controls (120, 121). Moreover, expression levels of 5-HT transporter protein are higher in psoriasis-involved vs. uninvolved skin (122). This line of evidence suggests stress-induced elevation in 5-HT can account for, at least in part, a mechanism by which stress negatively impacts psoriasis. However, low serum 5-HT in psoriatic patients was also reported (123), which could be due to those patients experienced psychological stress that is negatively associated with serum 5-HT levels (124–126). Both aggressive individuals and those with psychological stress also have lower serum levels of 5-HT (127, 128). Selective serotonin reuptake inhibitor increases serum levels of 5-HT (129, 130). In addition, serum 5-HT levels vary with patient’s age, with older patients having lower levels of 5-HT (131). Thus, variation of serum levels of 5-HT among the studies can be attributable to subjects’ age, psychological condition, and treatment.

The pathogenic role of 5-HT in psoriasis is complex, as its regulation of immune function varies depending on the specific serotonin receptors involved. For example. Selective inhibition of 5-HT2A receptor decreases the production of IFN-*γ*, a critical cytokine in psoriasis (107). Blockade of 5-HT3 receptor inhibits inflammation in rat (132). Similarly, inhibition of 5-HT7 receptor downregulates expression levels of the proinflammatory cytokines, IL-1β, IL-6, TNF-*α*, and IFN-γ in mice (133). In contrast, other study showed that activation of 5-HT2A receptor stimulated Tregs proliferation along with inhibition of Th17 differentiation and IL-17 production (134). UV irradiation-induced immunosuppression is also mediated by activation of 5-TH2A receptor (135). Additionally, 5-HT inhibits the production of IL-22, IL-6, and IL-17, possibly via 5-TH2B receptor *in vitro* (107, 136). Activation of 5-TH7 receptor either increases or inhibits inflammation depending on the types of cells and condition (137). The inflammatory effects of serotonin (5-HT) are both receptor subtype–specific and dose-dependent, reflecting its complex immunomodulatory profile (138). Therefore, further study is needed to clarify the pathogenic role of 5-HT in psoriasis.

### 3.3 Nerve growth factor and substance P

The skin is innervated by at least three types of nerve fibers, i.e., A, B, and C fibers, exerting sensation and regulation of cutaneous functions through secretion of neuropeptides and neurotrophins (139). In addition to upregulation of expression levels of nerve growth factor and its receptors in the skin (140), psychological stress increases circulating levels of nerve growth factor by over 80% (141). Nerve growth factor stimulates proliferation of neuron/non-neuron cells, including keratinocytes and fibroblasts, while inhibiting keratinocyte apoptosis, a feature of psoriasis (142, 143). Moreover, nerve growth factor induces inflammation via stimulation of degranulation and cytokine release from mast cells in the skin (143). Application of neutralizing nerve growth factor antibody decreases number of degranulated mast cells in the dermis and subcutaneous tissues of sonic stressed mice (139). Psoriatic skin expresses high levels of nerve growth factor and its receptors along with increased nerve innervation (144). In addition, nerve growth factor stimulates angiogenesis, upregulates expression levels of adhesion molecules, and activates T cells (143). The role of nerve growth factor in the pathogenesis of psoriasis is further supported by the evidence that inhibition of nerve growth factor receptor with either its blocker or its neutralizing antibody improves psoriasis (145). Hence, stress-induced increases in nerve growth factor can contribute, in part, to the pathogenesis of psoriasis.

Psychological stress also increases substance P in the brain (146), which then is transported to peripheral nerve terminals (147). Substance P and its receptors also express in various types of immune cells such as mast cells, neutrophils, eosinophils, T cells and monocytes. Studies showed that substance P induces infiltration of neutrophils in the skin and increases chemokine and chemokine receptor expression in primary mouse neutrophils. Moreover, substance P upregulates IL-2 expression in activated T cells in addition to stimulation of T cell proliferation and upregulation of the expression levels of macrophage inflammatory protein-1β (148). In addition, substance P upregulates IL-17A and IFN-*γ* production by human memory CD4(+) T cells, and promotes generation of bona fide Th17 cells and Th1/Th17 cells from non-Th17-committed CD4(+) memory T cells mediated by neurokinin 1 receptor. Substance P also stimulates the production of IL-1β, IL-6, TNF-*α*, and IL-23 production, and upregulates TNF-like 1A expression on monocyte surface (149). In peripheral blood mononuclear cells, substance P and IL-23 reciprocally regulate each other (150). These cytokines (TNF-α, IL-17 and IL-23) play a crucial role in the pathogenesis of psoriasis (151). Accordingly, their inhibitors have been utilized in the treatment of psoriasis. Thus, stress-induced increases substance P can also contribute, in part, to the pathogenesis of psoriasis.

### 3.4 Compromised epidermal permeability barrier function

Epidermal permeability barrier primarily resides in the stratum corneum. Psychological stress delays epidermal permeability barrier recovery in both murine and humans (152–154). Psychological stress-induced abnormality in epidermal permeability barrier is in part mediated by glucocorticoids because either RU-486 (a GC receptor antagonist) or antalarmin (a corticotropin-releasing hormone receptor antagonist) overcomes stress-induced abnormalities in epidermal structure and function (155). Compromised epidermal permeability barrier function can provoke cutaneous and systemic inflammation (156, 157), leading to the development of psoriasis. The pathogenic role of epidermal permeability barrier dysfunction in psoriasis is evidenced by several clinical observations. First, psoriasis is prone to develop on the body sites such as extensors, which are more vulnerable to disruption of epidermal permeability barrier. Second, alleviation of psoriasis can be achieved by improvement in epidermal permeability barrier function with occlusion or topical barrier-repair emollient (158–160). Koebner’s phenomenon is a good exemplar that disruption of epidermal permeability barrier provokes psoriasis. Additionally, topical emollient can prevent the relapse of psoriasis (161–163). Further, delayed epidermal permeability recovery was observed in both psoriasis-involve and -uninvolved skin (164). Taken together, this line of evidence indicates that psychological stress-induced epidermal permeability barrier dysfunction can account for an additional mechanism by which psychological stress negatively affects psoriasis.

Another noteworthy aspect is the disturbed sleep in patients with psoriasis, which can adversely affect both epidermal permeability barrier and immune function. The prevalence of sleep disorders is reported to be as high as 77% among patients with psoriasis, and their Pittsburgh Sleep Quality Index scores can be twice as high compared to controls (165), partly attributable psoriasis-related pruritus and psychological stress. Deprivation of sleep alone can adversely impact epidermal permeability barrier function (166, 167), consequently triggering and/or worsening psoriasis. Moreover, sleep deprivation also increases plasma IL-6, interleukin-1β, and TNF-*α* (166, 168). Hence, disturbed sleep is additional mechanism contributing to the adverse influence of psychological stress on psoriasis.

Additionally, as aforementioned, antidepressants such as fluoxetine and bupropion can increase the risk of psoriasis. Several cases of antidepressant-induced psoriasis have been reported (41–46, 169–171). More psoriatic patients than non-psoriatic individuals use antidepressants after anti-psoriatic treatment (172). Therefore, the risk of psychological stress for exacerbation/induction of psoriasis can be attributable, at least in part, to the use of antidepressants.

**Figure 1:**
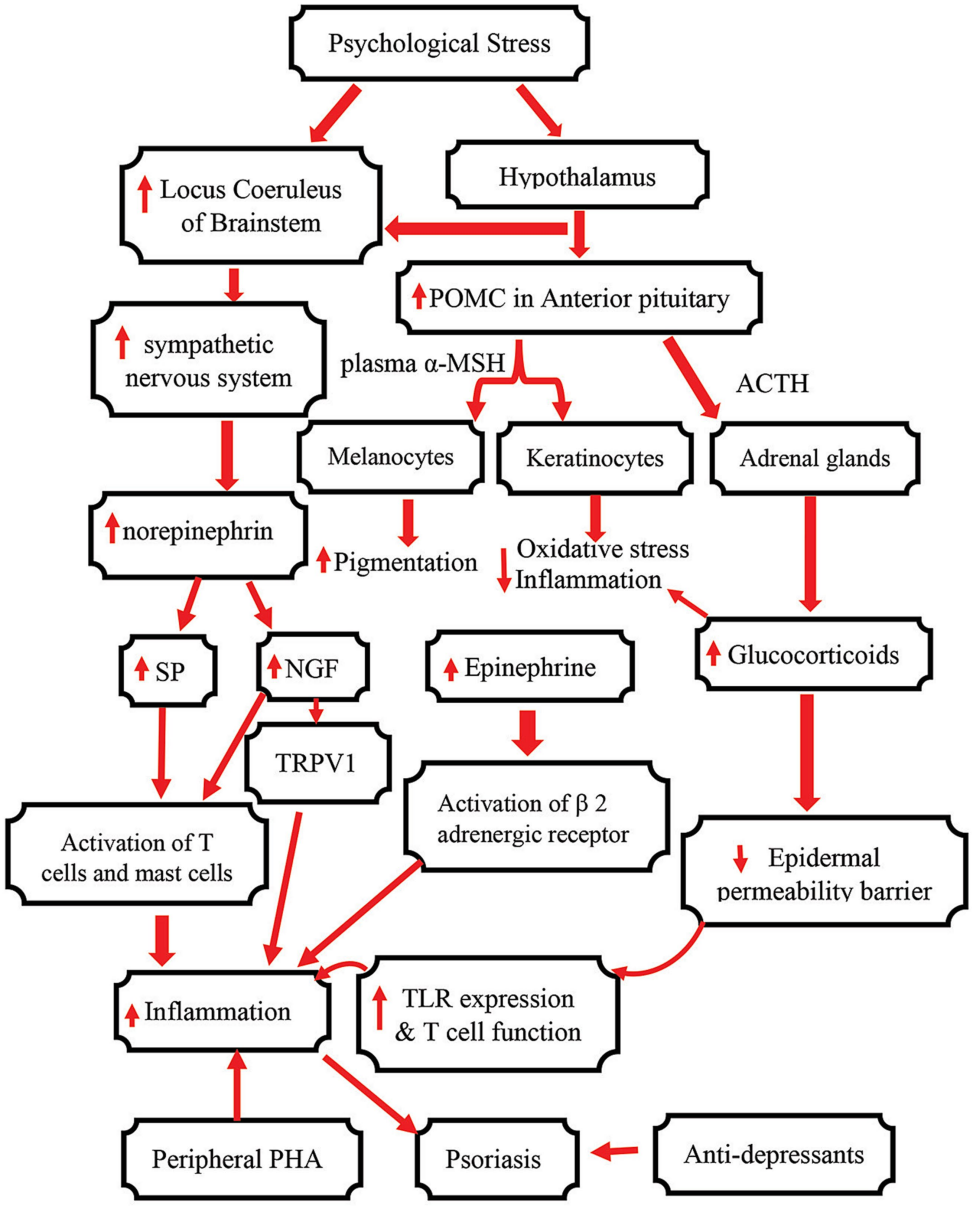
Schematic diagram showing the mechanisms by which psychological stress influences psoriasis. *α*-MSH, α-melanocyte stimulating hormone; ACTH, Adrenocorticotropic hormone; CRH, Corticotropin releasing hormone; POMC, Proopiomelanocortin; SP, substance P; NGF, nerve growth factor; GR, glucocorticoid receptor; TRPV1, transient receptor potential vanilloid 1.

In summary, psychological stress can exacerbate and/or predispose to the development of psoriasis, which is mainly mediated by central and peripheral PHA-axis (Figure 1). Effective treatment of psoriasis can mitigate psychological symptoms. Conversely, management of psychological stress, such as meditation, physical exercise, relaxation and hypnosis, can be beneficial for psoriatic patients with psychological disorders. However, the effectiveness of each regimen for psychological disorder varies with individual. Individualized plan for psychological intervention would be desirable. Because of the risk of antidepressants for psoriasis, caution should be taken when administering antidepressants to psoriatic patients.
